# Life against algorithmic management: a study on burnout and its influencing factors among food delivery riders

**DOI:** 10.3389/fpubh.2025.1531541

**Published:** 2025-04-15

**Authors:** Jian Dong, Guoyong Zhang, Lizhi Wu

**Affiliations:** ^1^School of Grammar and Law, Shandong University of Science and Technology, Qingdao, China; ^2^School of Medical Management, Shandong First Medical University, Jinan, China

**Keywords:** algorithmic management, food delivery platform, food delivery riders, burnout, influencing factor

## Abstract

**Background:**

With the rapid development of global digital economy, burnout among food delivery riders has become an important public health issue. Although burnout has been widely studied, research on burnout among food delivery riders, particularly the impact of algorithmic management on riders’ burnout remains limited. This study adopts a novel perspective on the intersection of algorithmic management and burnout, offering an in-depth examination of the burnout levels of food delivery riders under the strict control of algorithmic management and identifying its influencing factors.

**Methods:**

A survey of 953 food delivery riders was conducted using the Maslach Burnout Inventory-General Survey (MBI-GS). SPSS was used to conduct independent sample t-tests, one-way ANOVA, Pearson correlation, and multiple linear regression to investigate burnout status and identify factors affecting riders’ burnout.

**Results:**

The findings indicate that food delivery riders are experiencing moderate level of burnout, with Emotional Exhaustion, Depersonalization, and Reduced Personal Accomplishment as the primary dimensions. In the context of algorithmic management, key factors affecting riders’ burnout include gender, age, working years, ranking system, Punishment system, work rules, Work monitoring mechanism, workflow design, customer feedback, and restaurant preparation time.

**Conclusion:**

Under algorithmic management, burnout is prevalent among China’s food delivery riders and influenced by multiple factors. Individualized support, humane organizational systems, satisfied work mechanism, and supportive social environment can help lessen algorithmic management’s negative effects on food delivery riders and reduce their burnout. This study provides theoretical recommendations to protect occupational health of gig workers in platform economy, and offers valuable guidance for practical application.

## 1 Introduction

Driven by the rapid advancement of digital economy, the food delivery industry has undergone remarkable growth. In 2022, the global food delivery market was valued at approximately $1,100 billion, and it is projected to reach $1,510 billion by 2027 (1). As the central workforce in food delivery industry, food delivery riders have also experienced significant expansion. In China, as of 2024, 545 million people have used food delivery services, over 13 million riders are registered—a number that continues to increase. As a new occupation, food delivery riders have created significant economic value for the city, provided great convenience for citizens’ lives, and infused new vitality into city development, they are often praised as the ‘ferrymen of the city’ (2). However, such an important group has been under strict the control of algorithmic management (3, 4), the labor control imposed by algorithmic management exposes riders to varying levels of burnout, which not only harms their occupational health, but also leads to social problems that threaten social harmony and stability (1, 5). Currently, the burnout-related issues among food delivery riders have attracted global attention from scholars and practitioners, marking it as a critical public health issue, therefore, it is urgent and essential to research on the issues of burnout among food delivery riders. However, there is limited research on food delivery riders’ burnout, particularly the research on the impact of algorithmic management on riders’ burnout is even more scarce, which provides research space for our paper. To address the limitations of current studies, our paper adopts a novel perspective that integrates algorithmic management and riders’ burnout, and offers an in-depth examination of burnout levels of riders under algorithmic management, which is assessed using a burnout measurement questionnaire tailored to food delivery riders. On this basis, our paper employs a four-dimensional analytical framework—‘individual-organizational -occupational -social’ factors-to further identify the key factors contributing to riders’ burnout, and explore how these factors affect riders’ burnout. The findings are not only helpful to expand research field of occupational burnout, but also provide new insights for alleviating riders’ burnout, reducing the adverse effects of algorithm management, and ultimately improving the efficiency and quality of food delivery services.

### 1.1 Food delivery riders’ burnout

Burnout is a state of physical and emotional exhaustion caused by excessive demands on an individual’s energy, resilience, or resources at work (6). Through extensive research, Maslach (7) identified three core dimensions of burnout: emotional exhaustion, depersonalization, and reduced personal accomplishment. Emotional exhaustion is manifested in people feeling exhausted in energy or emotional resources and lacking motivation to work. Depersonalization refers to people adopting negative attitudes and behaviors toward those served at work. Reduced personal accomplishment is manifested in people’s decreased sense of competence and satisfaction at work, leading to negative self-assessment in one’s role (8). Prolonged burnout will not only damage people’s physical and mental health, causing symptoms such as headaches, depression, and anxiety, but also undermine people’s occupational health, manifested as decreased job satisfaction, frequent absenteeism, and increased turnover intention (9), ultimately reducing work efficiency and quality. As a new occupation, food delivery riders experience strict labor controls and high-intensity work arrangements from delivery platform (10), along with intense competition among peers (11), and limited societal respect and support (12). This puts riders in a prolonged state of tension, causing them to suffer tremendous physical and mental pressure at work (13, 14), ultimately leading to burnout. Once burnout occurs, food delivery riders may make mistakes at work, increasing the risk of traffic accidents (15), additionally, they may display aggressive behaviors, contributing to road violence and other social problems (16). Given these risks, conducting empirical research on burnout among food delivery riders is urgent and essential.

### 1.2 Algorithm management in food delivery platform

As the core mechanism of food delivery platform, algorithm management functions as a process of using algorithm technology to make management decisions (17), and execute management tasks (18). Algorithmic management emphasizes utilizing the powerful data processing capabilities of algorithms to intelligently allocate tasks and arrange workflows. It employs rational algorithmic logic to dynamically track, monitor, direct, and evaluate the work activities of food delivery riders throughout the entire process (19). Moreover, it continuously improves and optimizes the work process based on the collected data (20). Algorithmic management has fundamentally transformed work processes, management models, organizational structures, and operational rules of food delivery platform. It enables precise matching of labor supply and demand, improving platform management efficiency and service quality. Thus, algorithmic management is often regarded as ‘the most rational observer and manager’ (21, 22). However, as the research deepens, recent studies indicate that algorithmic management also creates a high-intensity, high-pressure work environment for food delivery riders (23, 24). To maximize profits, platform uses algorithmic management to standardize rules and workflows, cut labor costs, and impose strict controls over the work process (25). This may undermine riders’ labor rights, rest time, health, and autonomy (26), and threaten their dignity and privacy (27). As a result, food delivery riders become ‘algorithmically managed workers’ or ‘computed laborers’ (26). These conditions may provoke resistance among riders, leading to a series of social risks and ethical concerns.

### 1.3 Algorithm management and food delivery riders’ burnout

Burnout is a prominent adverse effect of algorithmic management on food delivery riders. Specifically, algorithmic management uses automated, data-driven approaches (28) to establish delivery rules and procedures, and enforce standardized and regulated control over riders’ work behavior (29). This damages riders’ autonomy, initiative, and sense of accomplishment (30), and triggers riders’ negative emotions like frustration, anxiety, and stress (31). Additionally, algorithmic management uses electronic monitoring systems to track riders’ activities throughout their work (32), such intensive ‘algorithmic surveillance’ makes riders feel oppressed at work and results in the phenomenon of ‘anxious freedom’, where riders feel trapped at work despite their perceived job flexibility (32, 33). Moreover, platforms frequently compress riders’ delivery time through algorithmic optimization, and force riders to rush to meet stringent deadlines, this persistent pressure reduces riders’ job satisfaction (29, 34, 35). Consequently, burnout caused by algorithm management makes riders feel dissatisfied, worried, anxious, and even fearful about the platform and its management practices (36, 37). In turn, riders may respond with counterproductive behaviors such as absenteeism, tardiness, or resignation to protest (21, 22, 25). Furthermore, algorithm management has a negative impact on the relationship between food delivery riders, customers and merchants, often leads to tensions, even physical and verbal conflicts between them (14).

### 1.4 Current study

Research has shown that the labor control imposed by algorithmic management often leads to varying levels of burnout among food delivery riders, resulting in a series of social problems, which has been observed in different countries. Therefore, it is crucial and urgent to study riders’ burnout within the context of algorithmic management. However, current studies have not sufficiently explored the impact of algorithmic management on riders’ burnout, lacking a comprehensive and systematic analytical framework. As a result, several critical questions remain unanswered: What role does algorithmic management play in causing burnout among riders? How severe is the burnout experienced by riders? What are the key factors contributing to riders’ burnout? How to alleviate riders’ burnout by optimizing algorithmic management practices? These unanswered questions offer research opportunities for our research.

To bridge the current research gaps, we integrate algorithmic management into the discourse on food delivery riders’ burnout, and utilize a tailored burnout questionnaire for riders to assess their burnout levels under algorithmic management. Moreover, we develop a comprehensive analytical framework that examines the influencing factors across four dimensions: individual, organizational, occupational, and social factors. Within the context of algorithmic management, we specifically hypothesize: (a) Individual factors, such as gender, marital status, age, working years, education, job type, and income, will influence riders’ burnout; (b) Organizational factors, such as ranking systems, punishment system, appeal system, work rules, insurance system, and performance evaluation system, will influence riders’ burnout; (c) Occupational factors, such as order dispatch mechanism, delivery route planning, delivery time calculation, work monitoring mechanism, workflow design, task assignment, will influence riders’ burnout; (d) Social factors, such as customer requirements, customer feedback, and merchant delivery speed, will influence riders’ burnout. Based on this framework, we use correlation and regression analyses to identify the key factors influencing riders’ burnout and explore how these factors cause riders’ burnout within the context of algorithmic management. This is helpful to deepen understanding of how algorithmic management may serve as a catalyst for riders’ burnout.

Through the study on burnout and its influencing factors among food delivery riders within the context of algorithmic management, this paper aims to deeply understand riders’ burnout status, identify potential drivers of burnout, and offer strategies to reduce the negative impacts of algorithmic management. Ultimately, our goal is to help riders adapt to their work and life with a more positive mindset. Additionally, we seek to increase awareness of riders’ real life in algorithmic era, call on people to recognize the difficulties faced by this group, advocate for the refinement of public health policies, and enhance social support for their occupational health.

## 2 Methods

### 2.1 Participants and procedure

This study focused on two main objectives: (1) to assess the level of burnout among food delivery riders under algorithmic management, and (2) to identify and analyze the key factors influencing burnout. To achieve these objectives, we designed a comprehensive questionnaire tailored to assess burnout among food delivery riders and its influencing factors. The questionnaire was designed for riders from various regions across China, without geographical restrictions to ensure sample representativeness. Data collection employed three methods: (1) collaborating with platform managers for random sampling with their approval; (2) contacting restaurant managers, and requesting them to invite riders to complete the questionnaire after picking up foods in-store; (3) working with a professional survey company to facilitate data collection through their specialized services. The entire data collection process spanned 3 months, with a total of 1,100 questionnaires distributed both online and offline. A total of 956 responses were received, giving a response rate of 87%. After excluding incomplete questionnaires, 953 valid questionnaires were retained, with an effective response rate of 99%. These 953 valid questionnaires constituted the final sample for analysis. To ensure the quality of survey, we conducted a small-scale preliminary survey to refine and optimize the questionnaire items. During the data collection phase, rigorous quality control measures, such as logical verification and manual inspection, were implemented. To ensure participants’ privacy, all food delivery riders participated anonymously and voluntarily, and they were fully informed of the purpose and procedures of the survey. Personal identifiers were removed from all questionnaires, and the study complied with the ethical standards of the Scientific Research Committee of Shandong University of Science and Technology.

### 2.2 Measures

#### 2.2.1 Measurement of demographic characteristics

This study examined seven demographic variables: gender, marital status, age, working years, education, job type, and monthly income. Gender reflects personality traits that could impact riders’ work philosophy and decision-making. Marital status indicates the riders’ living situation that may influence their sense of responsibility and job stability. Age offers insight into riders’ views on work and life. Working years indicates the riders’ work experience and proficiency. Education reflects the riders’ knowledge and values at work. Job type may affect the riders’ career development prospects. While income serves as an indicator of riders’ work performance. Given the close relationship between these variables and riders’ individual characteristics, we considered demographic characteristics as individual factors influencing burnout. And through questionnaire surveys and data analysis, we seeks to reveal how individual factors affect riders’ burnout.

#### 2.2.2 Burnout scale

Based on Maslach Burnout Inventory-General Survey (MBI-GS) and the specific characteristics of food delivery riders, we developed the Food Delivery Rider Burnout Questionnaire. The questionnaire comprises 15 items across three dimensions: Emotional Exhaustion (items 1–5): This dimension assesses the riders’ physical and mental exhaustion; Depersonalization (items 6–9): This dimension assesses riders’ detached attitudes toward customers, colleagues, and merchants; Reduced Personal Accomplishment (items 10–15): This dimension assesses riders’ sense of work achievement and job satisfaction. The questionnaire uses a 5-point Likert scale, ranging from 1 (never) to 5 (always), where higher scores indicate higher levels of burnout.

#### 2.2.3 Burnout influencing factors

In the context of Chinese cultural characteristics, Zeng (38) argued that burnout results from a complex interaction among‘individual-occupational-organizational’ factors. Meanwhile, Minling and Xiaoxiao (9) emphasized the combined impact of‘individual-social’factors as key drivers of burnout. Drawing on relevant studies from different countries concerning the impact of algorithmic management on food delivery riders, this study expands the two models by incorporating the unique features of algorithmic management and the specific job characteristics of food delivery riders. We propose that burnout among food delivery riders is influenced by the interaction of four dimensions: ‘individual-organizational-occupational-social’ factors. Based on this expanded model, we developed a questionnaire to examine the factors influencing burnout among food delivery riders (see Appendix 1 for details).

Individual factors include seven demographic variables outlined in Section 2.2.1, and are addressed in Questions 1–7 of the questionnaire.

In terms of organizational factors, we focus on identifying elements within algorithm-driven organizational management that may contribute to riders’ burnout. Previous studies have highlighted several critical issues: the excessive difficulty of promotion within ranking system would weaken riders’ work motivation (14, 16); overly strict punishment system would make riders feel depressed and dissatisfied (39, 40);an inadequate appeal system would make riders feel helplessness (41, 42); strict enforcement of work rules would make riders feel nervous and anxious (21, 22, 30); insufficient insurance coverage would increase risk cost of riders’ injuries (43, 44); and unreasonable performance evaluation system would provoke skepticism among riders (28, 45). Accordingly, organizational factors mainly include ranking system, punishment system, appeal mechanism, work rules, insurance system, and performance evaluation system, covered in Questions 16–21 of the questionnaire.

In terms of occupational factors, we focus on identifying work-related elements shaped by algorithmic management that may lead to riders’ burnout. Relevant studies have shown that: algorithm-driven order dispatch mechanism places intense pressure on riders to compete for orders (20, 46); discrepancies between algorithm-planned delivery routes and real-world scenarios create confusion and stress among riders (47); algorithm- calculated delivery time compresses riders’ delivery windows, forcing riders to race against time, and increasing the risk of traffic accidents (29, 48); all-round work monitoring mechanism harms riders’ work autonomy (21, 22, 49); standardized, monotonous and repetitive workflows designed by algorithms result in riders’ boredom and dissatisfaction (50, 51); the excessive workload assigned by algorithms results in physical and mental exhaustion among riders (52, 53). Accordingly, occupational factors include order dispatch mechanism, delivery route planning, delivery time calculation, work monitoring mechanism, workflow design, workload assignment, covered in Questions 22–27 of the questionnaire.

In terms of social factors, we focus on the impact of social actors, including customers and merchants, on riders’ burnout under algorithmic management. Relevant studies indicate that: in customer-centric algorithmic management, customers may place excessive demands beyond the riders’ capabilities or responsibilities, which makes riders feel angry and powerless (42, 54); customers’ negative feedback could result in platform-imposed penalties for riders, which makes riders adopt an indifferent attitude toward customers (55, 56); in time-centric algorithmic management, slow merchant preparation speed could make riders to be punished for delivery delay, which exacerbates conflicts between riders and merchants (46, 57). Accordingly, social factors mainly include customer demands, customer feedback, and merchant preparation speed, covered in Questions 28–30 of the questionnaire.

### 2.3 Data analysis

This study adopts two methods: descriptive analysis and inferential analysis, uses IBM SPSS Statistics 29.0 for data processing, and sets significance levels at *p* < 0.05. Descriptive statistics are first used to calculate the mean scores and standard deviations (SD) of the overall burnout scale and its subscales to evaluate the current burnout level among food delivery riders within the context of algorithmic management. Independent sample *t*-tests and one-way ANOVA are then used to explore whether individual factors significantly influence riders’ burnout. Pearson correlation analysis follows, examining the bivariate relationships between overall burnout levels and organizational, occupational, and social factors, and exploring the correlation between these factors and riders’ burnout in an algorithm-managed setting. Finally, multiple linear regression analysis is used, taking overall burnout as the dependent variable, and organizational, occupational, and social factors, along with their sub-dimensions, as independent variables, to examine the effects of these factors on riders’ burnout.

## 3 Results

### 3.1 Demographic information for samples

Among the 953 food delivery riders surveyed, 782 (82.1%) were male and 171 (17.9%) were female. Regarding marital status, 502 riders (52.7%) were married, 65 (6.8%) were divorced or widowed, and 386 (40.5%) were single. The age distribution showed that 14 riders (1.5%) were teenagers aged 18–20, 501 (52.6%) were young adults aged 21–30, 391 (41%) were prime adults aged 31–40, 39 (4.1%) were middle-aged adults aged 41–50, and 8 (0.8%) were older adults riders above 50, notably, the majority (93.6%) of riders were between 20 and 40 years old, indicating a predominance of young and middle-aged workers in this occupation. In terms of working years, 91% of riders have been in this occupation for less than 8 years. Most riders (720, or 75.6%) worked full-time, while 233 (24.4%) were part-time. The educational data showed that 571 riders (59.9%) held associate degree or below, reflecting a low educational attainment among riders. Income analysis showed that the majority (90.9%) of riders earned between 3,000 and 10,000 RMB per month, while a smaller group (45 riders, or 4.7%) earned less than 3,000 RMB, and 42 riders (4.4%) earned over 10,000 RMB. In conclusion, the study find that food delivery riders are predominantly male, and primarily young or prime adults. Most riders are engaged in full-time work, with relatively low educational backgrounds, and a significant proportion are married. Their job tenures tend to be short, and their monthly income ranges from 3,000 to 10,000 RMB.

### 3.2 Prevalence of burnout among food delivery riders

#### 3.2.1 Current situation of burnout among food delivery riders

Burnout among food delivery riders is measured using a five-point Likert scale, where higher scores indicate more severe burnout. Burnout levels are classified into three levels: low, moderate, and high, based on one-third of the total score. Scores below 2 indicate low burnout, scores between 2 to 4 indicate moderate burnout, and scores above 4 indicate high burnout. Table 1 presents the burnout levels and their distribution among riders. The results show that the overall burnout score is 3.19 ± 0.77, reflecting a moderate level of burnout. Across specific dimensions, the scores are as follows: emotional exhaustion (3.20 ± 0.99), depersonalization (3.19 ± 0.99), and reduced personal accomplishment (3.18 ± 0.96), all reflecting moderate levels of burnout. These findings suggest that food delivery riders generally experience moderate burnout. Further analysis shows that 73.9% of riders (704 individuals) experience moderate burnout, 21.9% (209 individuals) experience high burnout, and only 4.2% (40 individuals) experience low burnout. Among the three dimensions of burnout, reduced personal accomplishment has the largest number of riders with moderate burnout (634 individuals), followed by depersonalization (610 individuals) and emotional exhaustion (604 individuals).

**Table 1:** Number of burnout in various dimensions.

Dimensionality	High burnout		Moderate burnout		Low burnout		Mean burnout	Std. deviation
	Number of people	Percentage	Number of people	Percentage	Number of people	Percentage		
Emotional exhaustion	176	18.5%	604	63.3%	173	18.4%	3.2	0.99
Depersonalization	167	17.5%	610	64%	176	18.5%	3.19	0.99
Reduced Personal Accomplishment	154	16.1%	634	66.5%	165	17.4%	3.18	0.96
Overall burnout	209	21.9%	704	73.9%	40	4.2%	3.19	0.77

Data source: Self-made by the author.

#### 3.2.2 Relationship between food delivery riders’ individual characteristics and burnout situation

This study examines the impact of demographic differences on burnout, providing valuable insights into how individual factors contribute to burnout among food delivery riders. We use independent sample t-tests and one-way analysis of variance, obtaining the following key findings (Table 2): (1) Gender has a significant impact on riders’ burnout. Significant differences are found in overall burnout (*p* < 0.001), emotional exhaustion (*p* = 0.005 < 0.05), and reduced personal accomplishment (*p* < 0.001), and female riders (3.37 ± 0.63) experience higher burnout levels than male riders (3.15 ± 0.76). (2) Marital status does not significantly influence riders’ burnout. No significant differences are observed in overall burnout (*p* = 0.13 > 0.05), emotional exhaustion (*p* = 0.07 > 0.05), depersonalization (*p* = 0.11 > 0.05), or reduced personal accomplishment (*p* = 0.47 > 0.05). (3) Age has a significant impact on riders’ burnout, particularly in overall burnout (*p* = 0.01 < 0.05) and emotional exhaustion (*p* = 0.003 < 0.05). Young riders (3.27 ± 0.78), prime-age riders (3.11 ± 0.77) and old riders (3.46 ± 0.67) exhibit higher levels of burnout. (4) Working years has a significant impact on riders’ burnout, with significant differences observed in overall burnout (*p* = 0.02 < 0.05) and emotional exhaustion (*p* = 0.01 < 0.05), the longer the working years, the lower the burnout level. (5) Education does not significantly influence riders’ burnout. No significant differences are observed in overall burnout (*p* = 0.68 > 0.05), emotional exhaustion (*p* = 0.33 > 0.05), and depersonalization (*p* = 0.17 > 0.05). However, higher-educated riders’ burnout (3.63 ± 0.67; 3.22 ± 0.95) are more severe than that of lower-educated riders (3.14 ± 0.96) in reduced personal accomplishment. (6) Job type has no significant influence on riders’ burnout. No significant differences are found in overall burnout (*p* = 0.8 > 0.05), emotional exhaustion (*p* = 0.35 > 0.05), and reduced personal accomplishment (p = 0.47 > 0.05). (7) Income has no significant influence on riders’ burnout. There are no significant differences in overall burnout (*p* = 0.13 > 0.05), emotional exhaustion (*p* = 0.49 > 0.05), depersonalization (*p* = 0.06 > 0.05) and reduced personal accomplishment (*p* = 0.5 > 0.05).

**Table 2:** Relationship between riders’ individual characteristics and burnout situation.

Individual characteristics	Emotional exhaustion			Cynicism			Professional efficacy			Overall burnout		
	Mean burnout	Std. deviation	*p*-value	Mean burnout	Std. deviation	*p*-value	Mean burnout	Std. deviation	*p*-value	Mean burnout	Std. deviation	*p*-value
Gender												
Male	3.16	0.98	0.005	3.17	0.99	0.11	3.12	0.95	<0.001	3.15	0.76	<0.001
Female	3.40	1.02		3.31	0.99		3.40	0.96		3.37	0.63	
Marital Status												
Married	3.19	0.98	0.07	3.15	1.01	0.11	3.14	0.94	0.47	3.16	0.75	0.13
Divorced/Widowed	2.95	1.11		3.06	1.01		3.2	0.89		3.08	0.79	
Single	3.26	0.98		3.27	0.98		3.22	0.98		3.25	0.78	
Age												
18–20	3.17	0.71	0.003	2.89	0.97	0.11	2.04	0.92	0.07	3.04	0.68	0.01
21–30	3.28	0.99		3.26	0.99		2.43	0.82		3.27	0.78	
31–40	3.09	1		3.14	0.98		2.28	0.8		3.11	0.77	
41–50	3	0.83		2.92	1.12		2.2	0.87		2.99	0.6	
>50	3.83	0.53		3.34	0.91		1.77	0.85		3.46	0.67	
Working years												
<3 years	3.32	0.97	0.01	3.25	0.99	0.54	3.27	0.94	0.1	3.28	0.77	0.02
3–8 years	3.15	1		3.18	0.98		3.14	0.97		3.15	0.78	
9–13 years	3	0.94		3.1	1.09		3.04	0.91		3.04	0.73	
>13 years	2.76	1.24		2.95	1.5		2.8	1.2		2.83	1.03	
Education												
Graduate	2.8	0.97	0.33	2.67	1.1	0.17	3.63	0.67	0.04	3.1	0.62	0.68
Undergraduate	3.22	0.97		3.2	0.98		3.22	0.95		3.21	0.76	
Associate Degree or Below	3.2	1		3.19	1		3.14	0.96		3.17	0.78	
Job type												
Full-time	3.22	0.97	0.35	3.21	0.99	0.04	3.16	0.97	0.47	3.19	0.77	0.8
Part-time	3.15	1.07		3.16	0.99		3.22	0.91		3.17	0.79	
Income												
<3,000	3.3	1.08	0.49	3.39	1.02	0.06	3.31	0.83	0.5	3.33	0.82	0.13
3,001–5,000	3.28	1.02		3.31	0.95		3.24	0.91		3.27	0.76	
5,001–7,000	3.17	0.99		3.15	1		3.17	0.98		3.16	0.78	
7,001–10,000	3.15	0.96		3.17	1.01		3.13	0.98		3.15	0.77	
>10,000	3.2	0.81		2.91	1		3.06	0.91		3.04	0.6	

Data source: Results were compiled from the raw data.

### 3.3 Factors influencing burnout among food delivery riders

#### 3.3.1 Correlation analysis

On the basis of exploring the relationship between riders’ individual factors and burnout, the Pearson correlation analysis is further used to explore bivariate relationships between ‘organizational-occupational-social’ factors and riders’ burnout. The total burnout score, serving as a single composite measure, is used to examine its relationship with 15 influencing factors across organizational, occupational, and social dimensions. The detailed correlation coefficients are as follows (Table 3). For organizational factors, the total burnout score shows significant positive correlations with various organizational systems: ranking system (*r* = 0.521**, *p* < 0.001), punishment system (*r* = 0.533**, *p* < 0.001), appeal system (*r* = 0.503**, *p* < 0.001), work rules (*r* = 0.571**, *p* < 0.001), insurance system (*r* = 0.512**, *p* < 0.001), and performance evaluation system (*r* = 0.534**, *p* < 0 0.001). These findings suggest that stricter organizational systems are closely linked to elevated burnout levels among riders. Regarding occupational factors, positive correlations are observed between the total burnout score and: order dispatch mechanism (*r* = 0.345**, *p* < 0.001), delivery route planning (*r* = 0.522**, *p* < 0 0.001), delivery time calculation (*r* = 0.531**, *p* < 0 0.001), work monitoring mechanism (*r* = 0.546**, *p* < 0 0.001), workflow design (*r* = 0.534**, *p* < 0 0.001), and workload assignment (*r* = 0.529**, *p* < 0.001). This indicates that algorithm operational challenges significantly contribute to riders’ burnout. In terms of social factors, positive correlations are found between the total burnout score and key social factors, such as customer demands (*r* = 0.378**, *p* < 0.001), customer feedback (*r* = 0.596**, *p* < 0.001), and merchant delivery speed (*r* = 0.589**, *p* < 0.001). Notably, customer feedback shows the strongest positive correlation, highlighting the substantial impact of customer pressures on riders’ burnout. Overall, the analysis demonstrates that all 15 factors across the organizational, occupational, and social dimensions are positively associated with riders’ burnout. The strongest correlation was observed with customer feedback, indicating its dominant influence on burnout, while the performance evaluation system shows the weakest correlation, suggesting a relatively minor impact.

**Table 3:** Correlation analysis of burnout and its factors.

Factors affecting burnout	Correlation coefficient	Sig. (bilaterally)
Ranking system	0.521**	*p* < 0.001
Punishment system	0.533**	*p* < 0.001
Appeal system	0.503**	*p* < 0.001
Work rules	0.571**	*p* < 0.001
Insurance system	0.512**	*p* < 0.001
Performance evaluation system	0.324**	*p* < 0.001
Order dispatch mechanism	0.345**	*p* < 0.001
Delivery route planning	0.522**	*p* < 0.001
Delivery time calculation	0.531**	*p* < 0.001
Work monitoring mechanism	0.546**	*p* < 0.001
Workflow design	0.534**	*p* < 0.001
Workload assignment	0.529**	*p* < 0.001
Customer demands	0.378**	*p* < 0.001
Customer feedback	0.596**	*p* < 0.001
Merchant preparation speed	0.589**	*p* < 0.001

Data source: Self-made by the author.

#### 3.3.2 Multiple linear regression analysis

To further explore the impact of organizational, occupational, and social factors on riders’ burnout under algorithmic management, we develop a regression model using the total burnout score as dependent variable and the aforementioned factors as independent variables. The results of multiple linear regression analysis are summarized in Table 4. The adjusted R-squared value of model is 0.593, indicating that 59.3% of the variation in riders’ burnout could be explained by the model’s independent variables. The adjusted R-squared value exceeds 50%, indicating strong explanatory power. Additionally, the Durbin-Watson statistic is 1.923, which is close to the ideal value of 2, indicating no significant autocorrelation and satisfying the model’s independence assumption. The model’s overall *p*-value is <0.001, indicating that at least one of the independent variables significantly influences on the dependent variable (burnout). A further analyzing of the coefficients table shows that the *p*-values for organizational, occupational, and social factors are all <0.001, confirming their significant influence on riders’ burnout. Specifically, the regression coefficients for organizational, occupational, and social factors are 0.350, 0.292, and 0.338, respectively. All coefficients are positive, indicating that organizational, occupational, and social factors have significant positive impacts on riders’ burnout.

**Table 4:** Multiple linear regression analysis of burnout among riders.

Model	Non-standardized coefficient		Standard coefficient	*t*-value	Sig.	R square	Adjusted R square	Durbin-Watson	F
	B	Standard error	Beta						
(Constant)	0.706	0.069		10.282	<0.001	0.594	0.593	1.923	463.023 *p* < 0.001
Organizational factor	0.269	0.018	0.350	14.757	<0.001				
Occupational factor	0.236	0.020	0.292	12.082	<0.001				
Social factor	0.276	0.020	0.338	14.059	<0.001				
a. Dependent variable: Total score of burnout									

Data source: Self-made by the author.

To further analyze the influence of specific factors within each independent variable on riders’ burnout, we conducted multiple linear regression analyses for individual indicators. The results are as follows (Table 5): For organizational factors, the ranking system (*β* = 0.072, *p* = 0.021<0.05), punishment system (*β* = 0.086, *p* = 0.007<0.05), and work rules (*β* = 0.133, *p*<0.001), are significantly associated with riders’ burnout. This indicates that greater difficulty in ranking, stricter penalties, and more rigid work rules are associated with higher levels of burnout among riders. Regarding occupational factors, work monitoring mechanism (*β* = 0.135, *p* < 0.001) and workflow design (*β* = 0.092, *p* = 0.003 < 0.05), show significant associations with riders’ burnout. This indicates that extensive monitoring and repetitive workflows contribute more readily to riders’ burnout. In terms of social factors, customer feedback (*β* = 0.208, *p* < 0.001) and merchant preparation speed (*β* = 0.180, *p* < 0.001) are significantly associated with riders’ burnout. This indicates that higher frequency of negative customer reviews and merchant’s slow preparation are linked to higher burnout levels among riders.

**Table 5:** Multiple linear regression analysis of factors influencing burnout.

Factors affecting burnout	Unstandardized coefficients		Standardized coefficients	t	Sig.	Collinearity statistics	
	B	Std. error	Beta			Tolerance	VIF
Ranking system	0.049	0.021	0.072	2.306	0.021	0.415	2.412
Punishment system	0.059	0.022	0.086	2.691	0.007	0.394	2.541
Appeal system	0.029	0.022	0.041	1.347	0.178	0.424	2.357
Work rules	0.094	0.022	0.133	4.234	<0.001	0.406	2.460
Insurance system	0.007	0.022	0.010	0.304	0.761	0.383	2.610
Performance evaluation mechanism	−0.003	0.015	−0.005	−0.173	0.863	0.534	1.871
Order dispatch mechanism	0.013	0.014	0.024	0.923	0.356	0.606	1.650
Delivery process planning	0.037	0.021	0.053	1.755	0.080	0.434	2.304
Delivery time calculation	0.019	0.021	0.029	0.910	0.363	0.400	2.500
Work monitoring mechanism	0.095	0.022	0.135	4.409	<0.001	0.426	2.349
Workflow design	0.063	0.021	0.092	3.027	0.003	0.434	2.304
Workload assignment	0.030	0.021	0.044	1.401	0.162	0.403	2.484
Customer requirements	0.019	0.014	0.035	1.415	0.157	0.662	1.510
Customer feedback	0.143	0.020	0.208	7.202	<0.001	0.481	2.077
Merchant preparation speed	0.126	0.020	0.180	6.423	<0.001	0.508	1.969
a. Dependent Variable: Total score of burnout							

Data source: Self-made by the author.

## 4 Discussion

Our study offers a comprehensive analysis of burnout levels of food delivery riders in China, identifying various influencing factors of burnout under algorithm management. The findings indicate that 73.9% of riders experienced moderate burnout, with notable differences across three dimensions: emotional exhaustion, depersonalization, reduced personal accomplishment. Through independent sample T-tests, one-way ANOVA, and multiple regression analyses, key factors influencing riders’ burnout are identified, including gender, age, working years, ranking system, punishment system, work rules, work monitoring mechanism, workflow design, customer feedback, and merchant preparation speed. These influencing factors can be categorized into four dimensions: individual factor, organizational factor, occupational factor, and social factor. Our study enriches the current studies on burnout among food delivery riders, and provides valuable insights for refining algorithm management strategies on food delivery platform and alleviating occupational burnout among riders.

Analysis of individual factors shows that, firstly, gender is a key factor in food delivery riders’ burnout, which is consistent with previous studies (58, 59). Previous studies suggest that food delivery work is characterized by intense workloads, strict control, and demanding physical tasks, and traditionally perceived as “masculine,” and the structural constraints of algorithmic management further marginalizes female riders, exacerbating their burnout (60). Our study further confirms that female riders experience higher levels of burnout than their male counterparts. The possible reason is that women often shoulder heavier household responsibilities, increasing their physical and emotional exhaustion under the dual pressure of work and family. Moreover, physiological differences make women more susceptible to fatigue in food delivery work, while gender-specific safety concerns further exacerbate their anxiety. Secondly, age is another key factor influencing food delivery riders’ burnout, which is consistent with previous studies (61). Previous studies suggest that older riders tend to experience higher levels of fatigue (36). In contrast, our study indicates that young, prime-age, and old riders are more likely to experience burnout, compared to teenagers and middle-aged riders. The possible reason is that young riders, being new to the workforce, often struggle to adapt to the fast-paced demands of algorithmic management, leading to feelings of passivity and restriction and lack senses of achievement. Prime-age riders face heavy family obligations and financial pressures, such as child-rearing, aged care, and mortgage payments, the employment instability and income fluctuations caused by algorithm management can easily leave them physically and mentally exhausted (23). Old adult riders, facing declining health and reduced adaptability to new technologies, experience increased strain from high-intensity tasks and the pressure to master algorithmic tools, which contributes to their burnout. Thirdly, working years is a key factor influencing food delivery riders’ burnout, which is consistent with previous studies (62). Our study further confirms that riders with longer service duration exhibit lower levels of burnout. The possible reason is that over time, riders gain experience and adapt better to algorithmic management, allowing them to handle work challenges more effectively, thereby reducing their burnout levels. These findings suggest that support measures tailored to address the specific needs of riders based on gender, age, and working years are required. Providing gender-friendly supports, job stability initiatives, and career development programs and job training programs could significantly alleviate burnout and enhance the well-being of food delivery riders.

Analysis of organizational factors shows that, firstly, ranking system is a key factor in food delivery riders’ burnout, which is consistent with previous studies (11, 63). Previous studies suggest that the algorithm-based ranking systems are highly demanding, often placing riders in states of tension and anxiety during promotion or retention process, ultimately leading to a decline in motivation (11). Our study further confirms that as the difficulties of the ranking systems increase, so does the level of riders’ burnout. The possible reason is that the algorithmic ranking system is designed to make promotion difficult and demotion easy, requiring riders to invest more times and efforts to maintain or improve their rank and avoid demotion, these heavy workloads can leave riders physically and mentally exhausted, diminishing their motivation. Secondly, punishment system is another key factor in food delivery riders’ burnout, which is consistent with previous studies (5, 31, 34, 35). Previous studies suggest that algorithm-driven punishment systems tend to be overly harsh, often causing anxiety, depression, and insecurity among riders (40). Our study further confirms that harsher punishments are correlated with higher levels of riders’ burnout. The possible reason is that the punishment systems prioritize food delivery efficiency and customer satisfaction at the expense of riders’ needs and rights. As a result, riders may feel stressed and unfairly treated, leading to resistance and negative coping behaviors. Thirdly, work rule is a key factor influencing food delivery riders’ burnout, which is consistent with previous studies (30). Previous studies suggest that strict algorithm-based work rules often make riders overly cautious and fearful of mistakes (12). Our study further confirms that stricter work rules are associated with higher levels of riders’ burnout. The possible reason is that rigid work rules constrain how riders carry out their tasks, trapping riders within the algorithmic guidelines and restricting their ability to manage their work rhythm and methods. As a result, riders experience a strong sense of restriction and helplessness, with reduced work autonomy. These findings suggest that it is of great value to implement human-centered changes within ranking systems, protect riders’ rights within punishment systems, and enhance riders’ autonomy and satisfaction within work rules.

Analysis of occupational factors shows that, firstly, work monitoring mechanism is a key factor in food delivery riders’ burnout, which is consistent with previous studies (21, 22, 64). Previous studies suggest that algorithmic management uses comprehensive electronic monitoring for labor control, which leads to a sense of emotional exploitation among riders (65). Our study further confirms that the comprehensive, real-time monitoring on riders’ work processes significantly intensifies riders’ burnout. The possible reason is that algorithmic management uses advanced technological tools for pervasive monitoring of riders’ activities, effectively depriving them of work autonomy. The threat of punishment linked to this pervasive monitoring generates a sense of oppression, leaving riders in a persistent state of anxiety and stress. Moreover, the continuous monitoring, collection, and evaluation of personal data—such as riders’ behaviors, attitudes, and trajectories—could lead to perceived privacy violations, triggering dissatisfaction, anxiety, and helplessness among riders. Secondly, workflow design is a key factor in food delivery riders’ burnout, which is consistent with previous studies (50, 51). Previous studies suggest that algorithm-driven workflows are often simplistic, repetitive and monotonous, lacking diversities and challenges, which often results in boredom among riders (50, 51). Our study further confirms that simplistic, repetitive and monotonous workflows are more likely to lead to riders’ burnout. The possible reason is that algorithmic management system breaks down workflows into standardized, repetitive work steps to maximize food delivery efficiency, however, such a design also renders the work mechanical and boring, lacking freshness and challenge, which in turn reduces riders’ sense of achievement and satisfaction at work. These findings suggest that minimizing constant monitoring, increasing riders’ autonomy and flexibility, and strengthening privacy protection measures, could help alleviate riders’ burnout. Additionally, diversifying task assignments and implementing job rotation mechanism within workflow design are of great value, which could potentially reduce monotony and foster a more stimulating and fulfilling work environment.

Analysis of social factors shows that, firstly, customer feedback is a key factor in food delivery riders’ burnout, which is consistent with previous studies (47). Previous studies suggest that riders often experience stress due to customer reviews (39, 47). Our study further confirms that customers’ negative feedback significantly increase riders’ burnout. The possible reason is that in customer-centered evaluation system, algorithmic management prioritizes customer interests and places unconditional trust in customers’ feedback. As a result, riders frequently feel unfairly treated and deprived of professional respect, leading them to develop a sense of resentment and indifference toward customer. Moreover, algorithmic systems link customer feedback directly to riders’ performance evaluations, where negative reviews and complaints result in lower ratings, fewer orders, and reduced income. This punitive model increases riders’ anxiety (34, 35), compelling them to suppress their emotions and engage in excessive emotional labor during interactions with customers to avoid negative feedback. Secondly, merchant preparation speed is a key factor in food delivery riders’ burnout, which is consistent with previous studies (46). Previous studies suggest that delays in food preparation increases riders’ stress and strain their relationships with merchants (57). Our study further confirms that merchants’ slow food preparation speed significantly exacerbates riders’ burnout. The possible reason is that rider’s delivery time is precisely calculated by algorithms, slow food preparation speed disrupt the planned delivery timeline and work rhythm. As a result, riders are forced to complete deliveries within shorter time frames to avoid penalties for lateness during subsequent delivery stages. This increases riders’ time pressure and task difficulty, making riders feel nervous and anxious, further exacerbating conflicts between riders and merchants. These findings suggest that it is of great value to establishing an effective appeal system for riders within the customer feedback process, developing occupational health intervention programs, such as regular mental health counseling, emotional management training, as well as introducing a reminder mechanism for food preparation times and a pre-notification mechanism for food preparation delays.

This study has several limitations. Firstly, although the paper identifies various factors influencing riders’ burnout, it does not explore how these factors interact as a system to influence riders’ burnout collectively. Future research should explore the relationships and interactions between these factors to clarify the underlying systemic mechanisms of riders’ burnout. Secondly, the paper uses a cross-sectional survey design, primarily relying on self-reports and feedback within a specific social context, which may limit causal inferences between the variables. Future research could consider using quasi-experimental and longitudinal designs to track the long-term impact of algorithmic management on riders’ burnout, offering clearer insights into the causal relationship between burnout and algorithms. Lastly, due to different regional cultures, systems, and economic environments, food delivery riders’ burnout status may be different in other parts of the world. Given that this paper’s sample is limited to Chinese riders, the generalizability and applicability of the findings to other regions may be constrained.

## 5 Conclusion

Food delivery riders play a crucial role in modern cities around the world. However, under the stringent control of algorithmic management, riders find their lives caught in a persistent struggle between the dominance of algorithmic systems and their desire for personal autonomy (66, 67). On one hand, riders are compelled to comply with algorithmic labor controls to sustain their livelihoods (11); on the other hand, they engage in continuous resistance against algorithmic management to defend their labor rights (31). This dual dichotomy, often described as a ‘life against the algorithm’, frequently results in both psychological stress and physical exhaustion, leading to burnout among food delivery riders.

In this paper, we integrate algorithmic management into the discourse on food delivery riders’ burnout, and identify the key contributing factors by drawing on research findings from multiple countries. Building on this foundation, we conduct a survey of food delivery riders in China to examine the prevalence of burnout driven by algorithmic management. The results indicate that riders experience a moderate level of burnout. Based on the survey results, we further explore the key factors influencing riders’ burnout from four dimensions: individual, organizational, occupational, and social. Our findings indicate that, due to differences in regional cultures, institutional systems, and economic contexts, the prevalence and determinants of riders’ burnout may differ across countries. In the Chinese context, for instance, factors such as age, working years, and merchant preparation speed are found to significantly impact on riders’ burnout—factors that differ from the existing research from other countries. These may be viewed as context-specific phenomena unique to China. At the same time, through global comparison, our study also reveals several common factors consistent with findings from other countries. Specifically, gender (individual factors); ranking systems, punishment systems, and work rules (organizational factors); work monitoring mechanisms and workflow design (occupational factors); and customer feedback (social factors) are all found to significantly contribute to riders’ burnout. These findings highlight the shared global challenges in protecting the labor rights and well-being of food delivery workers, and underscore the urgent need for targeted interventions in the evolving landscape of the digital economy.

Based on these findings, we propose a series of targeted intervention measures. These measures include providing tailored supports based on gender, age, and work experience, as well as enhancing job security, ensuring job stability and offering career guidance. Furthermore, adopting a more human-centric approach in ranking systems, emphasizing the protection of riders’ rights within punishment mechanisms and optimizing work rules to enhance riders’ autonomy and satisfaction could alleviate burnout. Additionally, reducing excessive monitoring, strengthening privacy protections, diversifying task assignments, implementing job rotation, establishing appeal system, developing occupational health intervention programs and introducing reminder or pre-notification mechanism for food preparation times are also recommended measures.

These findings not only address the gap of current studies on algorithmic management and food delivery riders’ burnout, but also provide theoretical basis and policy recommendations for better protecting the occupational health of gig workers in the broader platform economy, considering the increasing digitalization of work and the precarization of labor relations in the food delivery sector worldwide. Additionally, these findings offer valuable insights for improving the efficiency and quality of delivery services on food delivery platforms.

## Data Availability

The data supporting the conclusions of this article are available upon reasonable request from the corresponding author.

## References

[1] Ruggeri M Zaki MG Vinci G. Towards social life cycle assessment of food delivery: findings from the Italian case study. Int J Life Cycle Assess. (2024) 29:1116–36. doi: 10.1007/s11367-024-02300-2, PMID: 40151768

[2] Jinhao S. Dual uncertain ties and the takeout riders’ emotional labor. Youth Stud. (2022) 2:14–25.

[3] Galière S. When food-delivery platform workers consent to algorithmic management: a foucauldian perspective. N Technol Work Employ. (2020) 35:357–70. doi: 10.1111/ntwe.12177

[4] Huang H. Algorithmic management in food-delivery platform economy in China. N Technol Work Employ. (2022) 38:185–205. doi: 10.1111/ntwe.12228, PMID: 40143556

[5] Zhang F Ji YJ Lv HT Zhang YQ Phil B Fan JL. Travel satisfaction of delivery electric two-wheeler riders: evidence from Nanjing. Transp Res A Policy Pract. (2022) 162:253–66. doi: 10.1016/j.tra.2022.06.001, PMID: 40151496

[6] Freudenberger HJ. Staff burn-out. J Soc Issues. (1974) 30:159–65. doi: 10.1111/j.1540-4560.1974.tb00706.x, PMID: 40143556

[7] Maslach CH Schaufeli WB Leiter MP. Job burnout. Annu Rev Psychol. (2001) 52:397–422. doi: 10.1146/annurev.psych.52.1.397 11148311

[8] Lizano EL Barak MM. Job burnout and affective well being: a longitudinal study of burnout and job satisfaction among public child welfare workers. Child Youth Serv Rev. (2015) 55:18–28. doi: 10.1016/j.childyouth.2015.05.005

[9] Minling C Xiaoxiao W. Job burnout: connotation, measurement and formation. Foreign Econ Manag. (2019) 8:86–99. doi: 10.16538/j.cnki.fem.2019.08.007

[10] Chen TX Tian DZ Deng PH Zhou E Huang JJ. Study on instant delivery service riders’ safety and health by the effects of labour intensity in China: a mediation analysis. Front Public Health. (2022) 10:1–12. doi: 10.3389/fpubh.2022.907474 PMC9260233 35812478

[11] Yu ZZ Treré E Bonini T. Emergence of algorithmic solidarity: unveiling mutual aid practices and resistance among chinese delivery workers. Media Int Australia. (2022) 183:107–23. doi: 10.1177/1329878X221074793, PMID: 40144045

[12] Sun P. Your order, their labor: an exploration of algorithms and laboring on food delivery platforms in China. Chinese J Commun. (2019) 12:308–23. doi: 10.1080/17544750.2019.1583676, PMID: 40101104

[13] Puram P Gurumurthy A Narmetta M Mor RS. Last-mile challenges in on-demand food delivery during COVID-19: understanding the riders’ perspective using a grounded theory approach. Int J Logistics Manag. (2022) 33:901–25. doi: 10.1108/IJLM-01-2021-0024, PMID: 35579975

[14] Zhan J Li YR Zhao Y. More reliance, more injuries: income dependence, workload and work injury of online food-delivery platform riders. Saf Sci. (2023) 167:106264–10. doi: 10.1016/j.ssci.2023.106264, PMID: 40151496

[15] Oscar OT Elisabeth R Narelle H. Risky business: comparing the riding behaviors of food delivery and private bicycle riders. Accid Anal Prev. (2022) 177:106820–12. doi: 10.1016/j.aap.2022.106820, PMID: 36108421

[16] Luca B Laura C Sofia P Luciano F Paolo N Dario C. Occupational safety and health of riders working for digital food delivery platforms in the city of Milan, Italy. Med Lav. (2024) 115:e2024035–10. doi: 10.23749/mdl.v115i5.16278, PMID: 39450630 PMC11562669

[17] Wilson HJ Daugherty PR. Collaborative intelligence: humans and AI are joining forces. Harv Bus Rev. (2018) 4:114–23.

[18] Rosenblat A Stark L. Algorithmic labor and information asymmetries: a case study of uber’s drivers. Int J Commun. (2016) 10:3758–84. doi: 10.2139/ssrn.2686227, PMID: 39618929

[19] Meijerink J Keegan A. Conceptualizing human resource management in the gig economy toward a platform ecosystem perspective. J Manag Psychol. (2019) 34:214–32. doi: 10.1108/JMP-07-2018-0277, PMID: 35579975

[20] Möhlmann M Zalmanson L Henfridsson O Gregory RW. Algorithmic management of work on online labor platforms: when matching meets control. Manag Inf Syst Q. (2021) 4:1999–2022. doi: 10.25300/MISQ/2021/15333

[21] Liu SS Pei JL Ge CM Liu XL Chen YF. Algorithmic management of online labor platforms: theoretical exploration and research prospect. J Manag World. (2022) 2:225–38. doi: 10.19744/j.cnki.11-1235/f.2022.0021

[22] Liu SS Pei JL Zhong CY. Is the platform work autonomous? The effect of online labor platform algorithm management on job autonomy. Foreign Econ Manag. (2022) 2:51–67. doi: 10.16538/j.cnki.fem.20200811.301

[23] Wu PF Zheng RS Zhao Y Li YX. Happy riders are all alike? Ambivalent subjective experience and mental well-being of food-delivery platform workers in China. N Technol Work Employ. (2022) 37:425–44. doi: 10.1111/ntwe.12243

[24] Sofía PP Amparo S Marcela I. The active production of consent for employment precarity and the euphemisation of coercion in platform economies: the case of food delivery riders. Econ Ind Democr. (2024) 45:1040–66. doi: 10.1177/0143831X231219465, PMID: 40144045

[25] Newlands G. Algorithmic surveillance in the gig economy: the organization of work through lefebvrian conceived space. Organ Stud. (2020) 42:719–37. doi: 10.1177/0170840620937900, PMID: 40144045

[26] Long LR Liang JJ Dong JN. The human resource management of gig workers in the platform economy: challenges and countermeasures. Human Resources Develop China. (2021) 10:6–19. doi: 10.16471/j.cnki.11-2822/c.2021.10.001

[27] Donato C Martin K. Platform-dependent entrepreneurs: power asymmetries, risk, and strategy in the platform economy. Acad Manag Perspect. (2021) 35:584–605. doi: 10.5465/amp.2019.0103

[28] Duggan J Sherman U Carbery R McDonnell A. Algorithmic management and appwork in the gig economy: a research agenda for employment relations and HRM. Hum Resour Manag J. (2020) 1:1–19. doi: 10.1111/1748-8583.12258

[29] Gregory K. My life is more valuable than this: understanding risk among on-demand food couriers in Edinburgh. Work Employ Soc. (2020) 35:316–31. doi: 10.1177/0950017020969593, PMID: 40144045

[30] Kathleen G Adam R Luke E Ruth M. Algorithmic control in platform food delivery work. For Soc. (2019) 5:1–15. doi: 10.1177/2378023119870041, PMID: 40144045

[31] Kellogg KC Valentine MA Christin A. Algorithms at work: the new contested terrain of control. Acad Manag Ann. (2020) 1:366–410. doi: 10.5465/annals.2018.0174

[32] Lin JL Chen CF. Power relations and value choices in the gig economy platform under algorithmic decision-making: a case study of food delivery platforms. J Southwest Minzu University. (2021) 12:153–7. doi: 10.3969/j.issn.1004-3926.2021.12.020

[33] Malin BJ Chandler C. Free to work anxiously: splintering precarity among drivers for uber and Lyft. Commun, Culture & Critique. (2017) 10:382–400. doi: 10.1111/cccr.12157, PMID: 40143556

[34] Yu SS Liu M Liu SS Liu TT. Research on the influence mechanism of algorithmic control on the gig workers’ turnover intention. Chinese J Manag. (2024) 8:1152–62. doi: 10.3969/j.issn.1672-884x.2024.08.005

[35] Yu D Zhang J Yun G. Delivery riders’ safety and delivery efficiency in on-demand food delivery industry: the moderating role of monitoring algorithms. Res Transp Bus Manag. (2024) 55:101143–14. doi: 10.1016/j.rtbm.2024.101143

[36] Zheng YB Ma Y Guo LX Cheng JC Zhang YL. Crash involvement and risky riding behaviors among delivery riders in China: the role of working conditions. Transp Res Rec. (2019) 37:10. doi: 10.1177/0361198119841028

[37] Sergio AU Sebastián R Mauricio O. The hidden cost of your ‘too fast food’: stress-related factors and fatigue predict food delivery riders’ occupational crashes. Int J Occup Saf Ergon. (2024) 30:825–34. doi: 10.1080/10803548.2024.2356997, PMID: 38853658

[38] Zeng SC Li X He XS Chen W. Chinese social workers’ turnover intention and its impacting factors. J Chongqing Technol Business University (Soc Sci Edition). (2019) 4:1–10. doi: 10.3969/j.issn.1672-0598.2019.04.001

[39] Wood AJ Graham M Lehdonvirta V Hjorth I. Good gig, bad big: autonomy and algorithmic control in the global gig economy. Work Employ Soc. (2019) 33:56–75. doi: 10.1177/0950017018785616, PMID: 30886460 PMC6380453

[40] Liu SS Yu SX Liu C. Promotion or suppression? Double-edged sword effect of algorithm control on online working hours of gig workers. J Bus Econ. (2023) 5:17–28. doi: 10.14134/j.cnki.cn33-1336/f.2023.05.002

[41] Zou KL Wang X. The deviation, restoration and reconciliation of the take-out delivery riders’labor relations with the control of algorithm. J Dalian University of Technol (Soc Sci). (2022) 3:70–9. doi: 10.19525/j.issn1008-407x.2022.05.008

[42] Lu Y Yang MM Zhu JH Wang Y. Dark side of algorithmic management on platform worker behaviors: a mixed-method study. Hum Resour Manag. (2024) 63:477–98. doi: 10.1002/hrm.22211, PMID: 40145126

[43] McKinlay A Mitchell G Bertenshaw C. A scoping review of risk factors and injuries affecting food delivery riders. Emerg Med Australas. (2022) 34:150–6. doi: 10.1111/1742-6723.13927, PMID: 35037394

[44] Daufenback V Bógus CM Rocha C Ribeiro EA. ‘We are invisible to society’: impacts of working conditions on food delivery workers’ health and quality of life during the COVID-19 pandemic. Saude E Sociedade. (2023) 32:1–17., PMID: 40136223

[45] Papakostopoulos V Nathanael D. The complex interrelationship of work-related factors underlying risky driving behavior of food delivery riders in Athens, Greece. Saf Health Work. (2021) 12:147–53. doi: 10.1016/j.shaw.2020.10.006, PMID: 34178391 PMC8209359

[46] Li SL Jiang LH. A new mode of labor time control and fake experience of freedom—a study on the labor process of takeout platform riders. Soc Stud. (2020) 6:91–112. doi: 10.19934/j.cnki.shxyj.2020.06.005

[47] Liu M Liu X Li L Zhou J Liu SS. Approach and avoidance mechanisms in the effects of algorithm transparency on the service performance of food-delivery riders. Curr Issue Tour. (2024) 7:1–15. doi: 10.1080/13683500.2024.2376208

[48] Chen L. Labor order under digital control: a study on the labor control of takeout platform riders. Soc Stud. (2020) 6:113–35. doi: 10.19934/j.cnki.shxyj.2020.06.006

[49] Salmon PM Bhawana KC Irwin BG. What influences gig economy delivery rider behaviour and safety? A systems analysis. Saf Sci. (2023) 166:106263–12. doi: 10.1016/j.ssci.2023.106263, PMID: 40151496

[50] Rani U Furrer M. Digital labour platforms and new forms of flexible work in developing countries: algorithmic management of work and workers. Compet Chang. (2020) 25:212–36. doi: 10.1177/1024529420905187, PMID: 40144045

[51] Yao PB. Technical subordination: reconstruction of the identification benchmark of workers in the algorithmic era. Lanzhou Acad J. (2022) 2:101–12.

[52] Nguyen-Phuoc DQ Mai NX Ho-Mai NT Nguyen MH Oviedo-Trespalacios O. What factors contribute to in-role and extra-role safety behavior among food delivery riders? Transport Res F: Traffic Psychol Behav. (2024) 102:177–98. doi: 10.1016/j.trf.2024.01.013

[53] Zhang ZD Guo JN Li H Wang HL. Challenge or hindrance? The impact of platform algorithmic stressor on digital gig workers’proactive service behavior. Adv Psychol Sci. (2024) 1:1768–85. doi: 10.3724/SP.J.1042.2024.01768

[54] Vanessa D Claudia MB Cecília R Esther AR. ‘We are invisible to society’: impacts of working conditions on food delivery workers’ health and quality of life during the COVID-19 pandemic. Saude E Sociedade. (2023) 1:1–17. doi: 10.1590/S0104-12902023220528en

[55] Gandini A. Labour process theory and the gig economy. Hum Relat. (2019) 72:1039–56. doi: 10.1177/0018726718790002

[56] Dong HM Zhong SQ Xu SX Tian JF Feng ZX. The relationships between traffic enforcement, personal norms and aggressive driving behaviors among normal e-bike riders and food delivery e-bike riders. Transp Policy. (2022) 114:138–46. doi: 10.1016/j.tranpol.2021.09.014

[57] Zheng WR. The obscured subordination: the key core of‘internet+labor Relationships’and its coordination strategy—taking fast delivery persons as the object of observation. Political Sci Law. (2024) 8:20–30. doi: 10.15984/j.cnki.1005-9512.2024.08.001

[58] Barzilay AR. Discrimination without discriminating? Learned gender inequality in the labor market and gig economy. Cornell J Law Public Policy. (2019) 28:545–67. doi: 10.2139/ssrn.3426676, PMID: 39618929

[59] Nguyen MH Pojani D Nguyen-Phuoc DQ Thi BN. What if delivery riders quit? Challenges to last-mile logistics during the covid-19 pandemic. Res Transp Bus Manag. (2023) 47:8. doi: 10.1016/j.rtbm.2022.100941 PMC9763215 38013801

[60] Sun P Zhao YC Zhang QY. Platforms, gender and labor: female food delivery couriers’ gender performativity. J Chinese Women’s Stud. (2021) 6:5–16. doi: 10.19857/j.cnki.nr.2021.06.001

[61] Wang C Zhou LH Ren QW. Research on traffic safety of takeaway delivery based on perception imbalance. J Safety Sci Technol. (2021) 8:163–6. doi: 10.11731/j.issn.1673-193x.2021.08.025

[62] Lachapelle U Carpentier-Laberge D Cloutier MS Ranger L. A framework for analyzing collisions, near misses and injuries of commercial cyclists. J Transp Geogr. (2021) 90:102937–12. doi: 10.1016/j.jtrangeo.2020.102937, PMID: 40151496

[63] Benta M. Psychosocial work environment and mental wellbeing of food delivery platform workers in Helsinki, Finland: a qualitative study. Int J Qual Stud Health Well Being. (2023) 1:1–19. doi: 10.1080/17482631.2023.2173336 PMC9897739 36730307

[64] Parent-Rocheleau X Parker SK Bujold A Gaudet MC. Creation of the algorithmic management questionnaire: a six-phase scale development process. Hum Resour Manag. (2023) 63:25–44. doi: 10.1002/hrm.22185, PMID: 40145126

[65] Wang W. The labor process of digital capitalism and emotional exploitation. Dent Econ. (2021) 2:15–22. doi: 10.16158/j.cnki.51-1312/f.2021.02.002

[66] Liu CX Friedman E. Resistance under the radar: organization of work and collective action in china’s food delivery industry. China J. (2021) 86:68–89. doi: 10.1086/714292

[67] Bronwyn F. Uneven mobilities: the everyday management of app-based delivery work in Germany. Mobilities. (2022) 5:695–710. doi: 10.1080/17450101.2022.2114842

