# Supplementary material for: Life against algorithmic management: a study on burnout and its influencing factors among food delivery riders
Source: Front Public Health. 2025 Apr 15;13:1531541. doi: 10.3389/fpubh.2025.1531541 (PMC12037544; doi:10.3389/fpubh.2025.1531541)
Supplement: Supplementary file 1 [file Table_1.DOCX]

## Appendix 1: Sample Demographics and Questionnaire

**1.Selection and Characteristics of the Sample**

This paper investigates the burnout of food delivery riders in China, with a specific focus on its prevalence and influencing factors.The questionnaire was randomly distributed among food delivery riders in China to ensure the authenticity and reliability of the sample data to the greatest extent. We examines the relationship between burnout and demographic variables, including gender, marital status, age, working years, education, job type, and monthly income. See Table A.

## Table A. Sample Demographics

| 填答者的基本信息 |
| --- |
| 1.Gender：（1）Male（2）Female |
| 2.Marital Status：（1） Married （2）Divorced/Widowed （3） Single |
| 3.Age：（1）18-20 （2）21-30（3）31-40 （4）41-50 （5）>50 |
| 4.Working years：（1）Less than 3 years （2）4-8 years（3）9-13years（4）>13years |
| 5.Education：（1）Graduate（2）Undergraduate（3）Associate Degree or Below |
| 6.Job type：（1）Full-time （2）Part-time |
| 7.Monthly income：（1）<3000¥（2）3001-5000¥（3）5001-7000¥（4）7001-10000¥（5）>10000¥ |

**2 Questionnaire design**

The questionnaire is structured into three sections. The first section collects basic demographic information, consisting of 7 questions (see Table A). The second section measures the burnout levels among food delivery riders,consisting of 15 questions,of which 1 to 5 is emotional exhaustion dimension, 6 to 9 is depersonalization dimension, and 10 to 15 is the reduced personal accomplishment dimension（See Table B）. The third section examines the factors influencing burnout, consisting of 15 questions,of which 16 to 21 focus on organizational factors, 22 to 27 focus on occupational factors, and 28 to 30 focus on social factors(See Table C).

## Table B. Questionnaire design：Burnout levels among food delivery riders

| **Serial number** | **Items** | **Never** | **Rarely** | **Sometimes** | **Often** | **Always** |
| --- | --- | --- | --- | --- | --- | --- |
| N1 | The food delivery works make me feel physically and mentally exhausted. | 1 | 2 | 3 | 4 | 5 |
| N2 | At the end of a day delivering works, I feel completely exhausted. | 1 | 2 | 3 | 4 | 5 |
| N3 | Waking up in the morning to face a full day of delivery works makes me feel deeply fatigued. | 1 | 2 | 3 | 4 | 5 |
| N4 | Delivering foods all day is very stressful for me. | 1 | 2 | 3 | 4 | 5 |
| N5 | When confronted with delivery works,I feel on the verge of breakdown. | 1 | 2 | 3 | 4 | 5 |
| N6 | Since I began this job, my interest in delivery work has steadily declined. | 1 | 2 | 3 | 4 | 5 |
| N7 | Since starting this job, I have become less enthusiastic for delivery compared to before. | 1 | 2 | 3 | 4 | 5 |
| N8 | I often question whether the work I do has any real meaning. | 1 | 2 | 3 | 4 | 5 |
| N9 | I am becoming increasingly indifferent to whether my work makes a meaningful contribution. | 1 | 2 | 3 | 4 | 5 |
| N10 | When challenges arise during delivery work, I find it difficult to address them effectively. | 1 | 2 | 3 | 4 | 5 |
| N11 | I feel incapable of making meaningful contributions to the food delivery platform. | 1 | 2 | 3 | 4 | 5 |
| N12 | I feel that I’m not good at or competent for the food delivery work. | 1 | 2 | 3 | 4 | 5 |
| N13 | I do not feel particularly happy when completing some of the tasks in my delivery work. | 1 | 2 | 3 | 4 | 5 |
| N14 | In my delivery work, I don’t feel that I have accomplished many meaningful or valuable things. | 1 | 2 | 3 | 4 | 5 |
| N15 | I am unsure whether I can effectively complete my tasks during the delivery process. | 1 | 2 | 3 | 4 | 5 |

## Table C. Questionnaire design：Factors influencing food delivery riders’ burnout

| **Serial number** | **Items** | **Strongly disagree** | **Disagree** | **Neutral** | **Agree** | **Strongly agree** | |  |
| --- | --- | --- | --- | --- | --- | --- | --- | --- |
| N16 | I feel that the platform’s ranking system is overly difficult, which undermines my enthusiasm for career advancement. | 1 | 2 | 3 | 4 | 5 | |  |
| N17 | I feel that the platform’s punishment system is overly strict,which makes me feel depressed and dissatisfied. | 1 | 2 | 3 | 4 | 5 | |  |
| N18 | I feel that the platform’s appeal system is inadequate, which  makes me feel helplessness. | 1 | 2 | 3 | 4 | 5 | |  |
| N19 | I feel that the platform’s work rules is overly strict, which  makes me feel nervous and anxious. | 1 | 2 | 3 | 4 | 5 | |  |
| N20 | I feel that the platform’s insurance coverage is insufficient, which increases the costs of my injury. | 1 | 2 | 3 | 4 | 5 | |  |
| N21 | I feel that the platform’s performance evaluation system is unreasonable, which makes me lose trust in the platform. | 1 | 2 | 3 | 4 | 5 | |  |
| N22 | I feel that the order dispatch mechanism exacerbates competition, which places intense pressure to me. | 1 | 2 | 3 | 4 | 5 | |  |
| N23 | I feel that the delivery routes planning mechanism does not align with the realities of delivery, leaving me feeling confusion and stress. | 1 | 2 | 3 | 4 | | 5 | |
| N24 | I feel that the delivery time calculating mechanism force me to race against time, putting my life at risk while working. | 1 | 2 | 3 | 4 | 5 | |  |
| N25 | I feel that the all-round work monitoring mechanism undermines my autonomy at work. | 1 | 2 | 3 | 4 | 5 | |  |
| N26 | I feel that the platform’s workflows are standardized, monotonous and repetitive, which makes me feel boring and  and dissatisfied at work . | 1 | 2 | 3 | 4 | 5 | |  |
| N27 | I feel that the platform’s workloads are excessive, which leads me to physical and mental exhaustion. | 1 | 2 | 3 | 4 | 5 | |  |
| N28 | I feel that the customers’ excessive demands make me feel angry and powerless. | 1 | 2 | 3 | 4 | 5 | |  |
| N29 | I feel that the customers’ negative feedback makes me adopt an indifferent attitude. | 1 | 2 | 3 | 4 | 5 | |  |
| N30 | I feel that slow food preparation by merchants increases the likelihood of friction and conflicts with them. | 1 | 2 | 3 | 4 | 5 | |  |
